# Obstructive Sleep Apnea, Oxidative Stress and Cardiovascular Disease: Lessons from Animal Studies

**DOI:** 10.1155/2013/234631

**Published:** 2013-03-03

**Authors:** Rio Dumitrascu, Joerg Heitmann, Werner Seeger, Norbert Weissmann, Richard Schulz

**Affiliations:** Department of Sleep Medicine, University of Giessen Lung Center, Klinikstrasse 33, 35392 Giessen, Germany

## Abstract

Obstructive sleep apnea (OSA) is an independent risk factor for cardiovascular (CV) diseases such as arterial hypertension, heart failure, and stroke. Based on human research, sympathetic activation, inflammation, and oxidative stress are thought to play major roles in the pathophysiology of OSA-related CV diseases. Animal models of OSA have shown that endothelial dysfunction, vascular remodelling, and systemic and pulmonary arterial hypertension as well as heart failure can develop in response to chronic intermittent hypoxia (CIH). The available animal data are clearly in favour of oxidative stress playing a key role in the development of all of these CV manifestations of OSA. Presumably, the oxidative stress is due to an activation of NADPH oxidase and other free oxygen radicals producing enzymes within the CV system as evidenced by data from knockout mice and pharmacological interventions. It is hoped that animal models of OSA-related CV disease will continue to contribute to a deeper understanding of their underlying pathophysiology and will foster the way for the development of cardioprotective treatment options other than conventional CPAP therapy.

## 1. Introduction

Obstructive sleep apnea (OSA) is a highly prevalent sleep-related breathing disorder presenting with the clinical hallmarks of snoring, witnessed nocturnal apneas, and excessive daytime sleepiness. It is caused by repetitive collapse of a narrow upper airway during sleep with the main predisposing factor being obesity [1]. Large-scale epidemiological studies have clearly shown that untreated OSA is an independent cardiovascular (CV) risk factor. Amongst others, severe OSA (i.e., with an apnea-hypopnea-index (AHI) exceeding 30 per hour of sleep) can contribute to the emergence of arterial hypertension, heart failure, stroke, and pulmonary hypertension [2–4]. In addition, otherwise healthy OSA patients can already display more subtle CV changes such as endothelial dysfunction and vascular remodelling [5, 6].

Based on data obtained in patients with OSA, it is currently believed that sympathetic activation, inflammation, and oxidative stress play major roles in the pathophysiology of OSA-related CV diseases [7–10]. However, due to various reasons, the possibilities to conduct further human research into the relationship between OSA and CV diseases are limited. First, OSA patients often present with confounding factors increasing *per se* CV risk such as obesity, concomitant metabolic disease, and smoking. Second, CV diseases in OSA patients often need many years to develop in order to be diagnosed clinically. Third, it is difficult to perform more invasive experimental procedures in these patients. 

**Figure 1:**
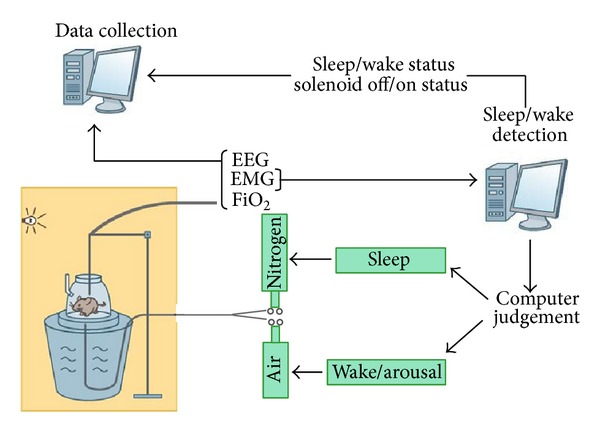
Schematic representation of the mouse/rat model simulating OSA-associated CIH. Animals are housed in plexiglass chambers and are repetitively exposed either to room air (i.e., 21%  O_2_) or nitrogen (i.e., hypoxia). Exposure to CIH may be synchronized to the sleep-wake cycle by simultaneously measuring EEG and EMG activities of animals.

These drawbacks may be overcome by using animal models of OSA. In most animal studies, only the cyclical pattern of hypoxia characteristic of OSA is simulated [11] (Figure 1). For this purpose, animals are housed in a chamber and cyclically exposed either to normoxia/hypoxia or room air in a computer controlled manner. 

Depending on animal species, type of stimuli, and technical approaches, there are many animal models described in the literature. The duration (from 30 seconds up to 30 min) of hypoxic exposure generally varies indirectly to the frequency of events (2 to 120/hour) [12–21], and this issue may contribute to discrepancies in the literature. However, the standard animal model is represented by chronic intermittent exposure of rodents to an FiO2 nadir of 6–10% for 30 sec to 1 min which results in oxyhemoglobin desaturation of about 60% to 80% (Figure 1).

It should be recognized that the above-mentioned experimental conditions primarily mimic severe forms of human sleep apnea with AHI >30/h. Furthermore, some of the human OSA characteristics are not highlighted in this experimental model. For example, the model fails to reproduce upper airway occlusion, intrathoracic pressure swings, arousals, and hypercapnia. On the other hand, reproducing a single characteristic of clinical OSA by exposure to cyclical hypoxia appears to be relevant in order to induce pathophysiological changes similar to clinical manifestations of patients suffering from OSA. Thus, animals exposed to CIH develop after 3 to 5 weeks pathological changes similar to those found in OSA patients such as endothelial dysfunction, atherosclerosis, arterial hypertension, pulmonary hypertension, and heart failure. Regardless of these considerations, other more complex models have also been developed which additionally reflect the nonhypoxic stimuli linked to OSA and may thus give a more realistic picture of the human disease [22, 23]. 

 In the present paper, we will discuss the animal models of OSA and their respective contributions to a deeper understanding of the CV consequences of OSA. Within this frame, we will focus on oxidative stress as the most relevant intermediary pathogenetic mechanism. The CIH exposure induces tissue deoxygenation followed rapidly by tissue reoxygenation leading to ROS formation. 

A few ROS-generating systems are described in the literature including NADPH oxidase (NOX), xanthine oxidase, mitochondrial chain, and uncoupled nitric oxide synthase (NOS). Probably, NOX is the most important enzyme in the setting of CIH as will be discussed in the following sections. The deleterious effects of ROS on the CV system are primarily exerted by unspecific oxidation of biological compounds (DNA, protein, lipid, etc.) and modulation of specific signalling pathways (i.e., redox signalling). In addition, ROS may activate the sympathetic nervous system and enhance inflammatory processes thus acting as master regulators in the pathogenesis of OSA-associated CV diseases.

## 2. Endothelial Dysfunction

Otherwise healthy, nonsmoking OSA patients have been shown to suffer from endothelial dysfunction, that is, a reduction of endothelial-dependent vasodilation, which may be relieved by effective continuous positive airway pressure (CPAP) therapy [6, 24]. Animal studies clearly suggest that the endothelial dysfunction characteristic of OSA is the result of an increased oxidative stress with reduced bioavailability of nitric oxide (NO). 

Rats exposed to chronic intermittent hypoxia (CIH) exhibit a reduced vasodilation in response to infusions of the NO liberator acetylcholine and reduced vasoconstriction following NO synthase inhibition [25]. Furthermore, treatment of CIH-exposed rats with the superoxide dismutase (SOD) mimetic tempol restores vascular reactivity [26]. Investigations of *in vitro* vasoreactivity of isolated coronary and cerebral arteries from mice have found that an activation of NOX is probably responsible for the occurrence of endothelial dysfunction in response to CIH [27]. 

Similar findings were reported by Liu et al. [28] describing erectile dysfunction in CIH rats due to increased ROS production. In this model, NOX is activated, NOS activity is impaired, and ROS production is increased. Furthermore, treatment of animals with apocynin (a selective NOX inhibitor) normalizes NOX and NOS activities and restores the erectile function. Recent clinical and experimental data suggest that xanthine oxidase-dependent ROS production also induces endothelial dysfunction in muscular skeletal arteries [29, 30] and that xanthine oxidase inhibition by allopurinol reverses this phenomenon.

## 3. Atherosclerosis

Endothelial dysfunction is a precursor lesion for atherosclerosis. Consequently, OSA patients display increased common carotid artery-intima media thickness when compared with matched controls without sleep-disordered breathing [5]. Furthermore, CPAP therapy leads to a decrease of this noninvasive marker of atherosclerosis [31]. Animal studies support the concept that the enhanced atherosclerosis known to occur in OSA is due to increased vascular inflammation and lipid peroxidation in response to oxidative stress.

 In this context, mice exposed to intermittent hypoxia exhibit increased leukocyte adhesion in their cortical venular microcirculation [32]. Another study reported increased flux of leukocytes rolling, a number of rolling leukocytes, and a number of adherent leukocytes in colonic venules following 3 hours of recurrent obstructive apneas in rats [33]. Of note, ROS may act as proinflammatory triggers by inducing NF-*κ*B and subsequently the expression of proinflammatory cytokines such as interleukin-6, tumor necrosis factor alpha and C-reactive protein [34]. 

In addition, they may exert proatherogenic effects by increasing lipid peroxidation as shown in a mouse model of OSA [35]. In accordance with these assumptions, direct evidence has been obtained in mice that CIH leads to the formation of atherosclerotic lesions. Exposure to CIH for 12 weeks induced the development of atherosclerotic plaques, but a concomitant high-cholesterol diet was necessary for that effect to occur [36].

## 4. Arterial Hypertension

Arterial hypertension is the most frequent CV complication of OSA and there is a significant dose-response relationship between the AHI and the odds ratio for developing arterial hypertension [37]. Animal studies have shown that various vasoactive mediator systems may be responsible for the pressor effect of CIH and that this is primarily mediated through an augmentation of carotid chemoreflex function. 

In a series of experiments, Flechter et al. were among the first to show the importance of the sympathetic nervous system in this context. They demonstrated that surgical denervation of peripheral chemoreceptors prevented the increase in arterial blood pressure in response to CIH. Adrenal demedullation and chemical denervation of the peripheral sympathetic nervous system by 6-hydroxy dopamine also prevented the increase in blood pressure [38, 39]. 

Carotid chemoreflex sensitization caused by CIH may also be due to angiotensin-II-induced activation of NOX with subsequent production of ROS. Chronic exposure of rats to CIH results in elevation of plasma renin activity, and pharmacological inhibition of the renin-angiotensin-aldosterone system attenuates CIH-induced arterial hypertension [40]. Similar effects can be observed after ascorbic acid (an antioxidant vitamin [41]), tempol [42], and apocynin [43].

Finally, data from our laboratory show that NOX knockout blocks the development of arterial hypertension in response to CIH [44]. As a more direct evidence, gene transcription of NOX subunits has been found to be upregulated in the carotid body in response to CIH [45]. 

The current concept is that CIH activates HIF-1*α* thereby enhancing NOX2 transcription and ROS production [46]. On the other hand, it decreases HIF-2*α*-dependent SOD activation and thus leads to a reduced clearance of ROS [47]. Importantly, the ROS formed within the carotid body may exert their pressor effects by enhancing central sympathetic activity [48]. 

Endothelin-1 is another vasoactive mediator which is strongly upregulated in the carotid body in response to CIH and receptor antagonism by bosentan abolished abnormal chemosensitization [49, 50]. In accordance with these findings, other groups have found that systemic administration of endothelin receptor antagonists to rats/mice prevents the increase of blood pressure during CIH [51, 52].

In contrast to angiotensin and endothelin-1, NO inhibits carotid body chemosensitivity [53]. Rats exposed to CIH express less neuronal NO synthase [54], and stimulation of NO production by L-arginine restores the carotid body chemosensitivity [53] thus suggesting that an impaired bioavailability of NO might be responsible for enhanced carotid chemoreflex sensitivity under CIH.

It should be mentioned that the OSA-associated arterial hypertension probably results not only from increased carotid chemoreflex but also from decreased baroreceptor activity. Finally, locally acting processes in peripheral blood vessels as discussed in Section 2 (i.e., reduced NO bioavailability) may play significant roles.

## 5. Pulmonary Hypertension

20–30% of untreated OSA patients suffer from pulmonary arterial hypertension. It was first thought that this phenomenon is restricted to patients with pulmonary co-morbidities such as COPD, but it is now widely accepted that OSA itself can lead to pulmonary hypertension [55]. Up to date, this aspect of OSA-associated CV morbidity has been less extensively investigated in animal models. A histomorphometric study showed that mice exposed to CIH develop characteristic features of pulmonary hypertension such as elevated pulmonary artery pressure, right ventricular hypertrophy, and muscularization of small pulmonary arteries [56]. Quite similar observations were later reported by another group [13]. Furthermore, it was demonstrated that NOX knockout mice are protected against the development of CIH-associated pulmonary hypertension [57]. A more recent study showed that pulmonary vasodilatory capacity is impaired under conditions of CIH and that this is related to increased vascular superoxide anion production [58]. Thus, the same pathogenetic mechanisms which have been proposed for acute hypoxic pulmonary vasoconstriction [59] may be operative in OSA-associated pulmonary hypertension. 

## 6. Heart Failure

OSA patients are also at increased risk for the development of chronic heart failure. This may be due to OSA-related arterial hypertension, coronary artery disease, and the direct negative inotropic effects of breathing against an occluded upper airway [60]. Experimental models of OSA support the notion that CIH negatively affects left ventricular (LV) function and that oxidative stress is an important mediator of myocardial damage.

Exposure of dogs to CIH induces LV hypertrophy and a decrease in ejection fraction [61]. Experimental CIH in rats/mice results in myocardial remodeling with myocyte hypertrophy and interstitial fibrosis finally leading to LV dysfunction [62, 63]. Moreover, these studies suggest that an increased myocardial oxidative stress plays a significant role in this context. Chen et al. observed a significant inverse relationship between LV function and the myocardial content of lipid peroxides [62]. Furthermore, myocardial NOX subunit expression is increased in response to CIH [64, 65], and NOX knock-out mice are obviously protected against the development of LV dysfunction in response to CIH [63]. Finally, treatment of mice with allopurinol significantly attenuates myocardial changes induced by CIH [66]. The exact mechanisms by which ROS induce LV dysfunction are not known, but it is largely accepted that oxidative stress causes a cytotoxic tissue injury by increased lipid peroxidation, protein oxidation, and direct DNA damage leading in this way to apoptosis, necrosis, and abnormal tissue repair processes.

## 7. Conclusions

**Figure 2:**
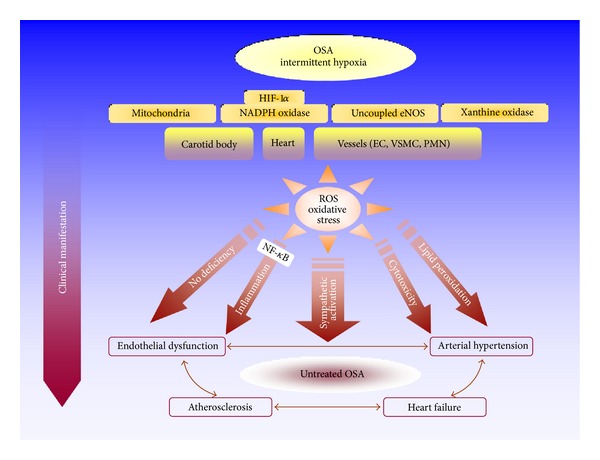
Simplified model of oxidative stress as the central pathogenetic pathway in OSA-associated CV diseases as suggested by animal studies. OSA-associated intermittent hypoxia activates NOX and other ROS-producing enzymes in the carotid body, the heart, and the vessels (PMN: polymorphonuclear neutrophils, EC: endothelial cells, VSMC: vascular smooth muscle cells). The resulting radical flux exerts direct cytotoxic effects, decreases NO bioavailability, enhances lipid peroxidation, increases sympathetic activity, and activates the proinflammatory transcription factor NF-*κ*B. Finally, these changes lead to the well-known clinical manifestations of OSA in the CV system.

In this paper, we have briefly summarized the current state of knowledge about the pathophysiology of OSA-related CV diseases as provided by basic research conducted in animal models of OSA. Almost the complete clinical spectrum of CV diseases known to occur in humans with OSA has been replicated in animals subjected to CIH. The common result of these studies is that an increased oxidative stress, mostly derived from an activation of NOX, seems to play a key role in the development of OSA-associated CV diseases (Figure 2). 

It is anticipated that animal studies will continue to enhance our understanding of the pathogenesis of OSA-related CV diseases for instance by investigating knock-out and genetically engineered mice or by performing selective pharmacological interventions. In this way, the fundamental molecular pathways linking OSA to CV diseases may be identified and new cardioprotective treatment options may emerge for the relatively large proportion of OSA patients unable to tolerate CPAP therapy. 

## Abbreviations

AHI: Apnea-hypopneaindex
CIH: Chronic intermittent hypoxia
CPAP: Continuous positive airway pressure
CV: Cardiovascular
LV: Left ventricular
NO: Nitric oxide
NOS: Nitric oxide synthase
NOX: NADPH oxidase
OSA: Obstructive sleep apnea
ROS: Reactive oxygen species
SOD: Superoxide dismutase.
